# The Duties of Medical Men to Medical Societies

**Published:** 1887-02

**Authors:** 


### Editorial.

### THE DUTIES OF MEDICAL MEN TO MEDICAL SOCIETIES.

The inaugural address delivered by Dr. Virgil O. Hardon upon assuming the office of President of the Atlanta Society of Medicine was one which is deserving of more than a passing notice, since it dealt in an earnest and thoughtful manner with a phase of professional duty which is too apt to escape recognition by the general practitioner of medicine. The meetings of medical societies are commonly looked upon rather in the light of social gatherings, where the physician may meet his professional brethren to discuss with them items of medical news, to recount interesting experiences and thus to while away a leisure hour and break up the monotony of daily professional toil. It is usually considered a matter of individual option whether he shall associate himself with such organizations, or, having so associated himself, whether he shall take an active part in their deliberations. Hence it is somewhat startling to see forcibly presented, as a "solemn obligation," the duties of joining medical societies, of participating in their proceedings, and, through the publication of their proceedings, of contributing to the literature of medical science. In a clear, logical and forcible manner the speaker argued these points, and at the conclusion of his address, we are satisfied that none of his hearers were disposed to gainsay the truth of his propositions.

We regret that we have not space to give this address in full, and no synopsis can do it justice. It was replete with terse, vigorous reasoning and contained much original thought expressed in characteristic epigrammatic style. We can only give a few extracts which will show the tenor and the style of the address. In speaking of the benefits of medical societies to the individual, it was said : "The tendency of the medical mind is to run into one or the other of two extremes—either to degenerate into blind, unreasoning routine or to soar into the realms of fanciful transcendentalism. The one results in the production of the intellectual myope who knows nothing beyond the narrow range of his own near-sighted vision ; the other produces the reverse of this, the man whose hypermetropic mental sight can appreciate only the distant abstract. Both are equally unfit for good professional or scientific work. To combat these tendencies, no agency is so effectual as the free discussion and comparison of individual opinions and experiences." "Individuality must be lost sight of, the personal equation must be eliminated, before a scientific principle can represent absolute truth."

Referring to the duty of the physician to his fellow-practitioners, the speaker said: "There is no man whose field is so limited that he cannot extract from it something which will be valuable to his fellow-practitioners. It is not necessary to be a Gross, a Spencer Wells or a Ricord in order to be an observer. The report of a successful ovariotomy is of far less value than the report of a successful method of treating infantile diarrhœa. The most common-place and every-day diseases furnish abundant field for observation and study. Who, for instance, can explain the true pathology of whooping cough, or tell how to treat it successfully? He who shall accomplish this will do a far greater good than he who performs an hundred consecutive laparotomies without a death."

In connection with the duty of giving to the world the results of individual research, he said: "A vast machine may lie idle for lack of a single bolt or pinion; and so a single fact, deduced by one of us from our own study and observation, if given to the world, may prove to be the missing part which is alone lacking in the perfection of some great truth."

These three extracts represent the three lines of the argument of the address, viz.: the duty of the physician to his patients in availing himself of every means of perfecting his medical knowledge, the duty of the physician to his fellow-practitioners in giving to them the benefit of his own observation and experience, and the duty of the physician to posterity in contributing to the advancement of medical science. None of these duties are completely fulfilled by him who fails to associate himself with medical societies.

In these days of professional quackery and of unethical competition for gain, it is gratifying to listen to views which aim at the establishment of so high a standard of professional duty, and which form so strong a plea for honest, conscientious work. It is to be regretted that this address could not have had, as it deserved to have, a larger circle of hearers than could be furnished by a local medical society. An adoption of these views and their practical application would wondrously enhance the usefulness of every medical society in the land, and would thus unquestionably accomplish far-reaching good.

In conclusion, we would congratulate the Atlanta Society of Medicine upon the selection of a presiding officer whose views of professional duty are of so elevated and worthy a character.

### ITEMS.

DR. ROBERT BATTEY, of Rome, is spending the winter in Florida.

SEVERAL cases of small-pox have been reported from Murray county in this State.

THE January number of *Scribner's Magazine* is gotten up in very handsome style, and its contents will afford its readers much pleasure. It is replete with interesting papers from several of America's most popular writers.

THANKS.—We are indebted to Dr. George C. Hummel, of Savannah, for an invitation to the annual supper of the Georgia Medical Society, which was given at the Pulaski House January 4th. This is the oldest medical society in the State, having been in successful operation since 1804.

THE ECLECTIC.—The January number of this sterling magazine begins a new volume, the forty-fifth. The *Eclectic* gives to its readers a reflex of foreign periodical thought, and is the only monthly periodical of its kind issued. It should receive the support of our home readers who desire to know what transpires in the world of foreign thought.

TO THE MEMBERS OF THE MEDICAL ASSOCIATION OF GEORGIA. Dr. J. McF. Gaston, chairman of the Section of Surgery for the Fifth Congressional district, requests us to ask those having cases of interest in surgery to furnish reports of the details if practicable; or, if this is not convenient, to give notes of the most important points as early as may be practicable. Those who may be able to prepare papers giving their surgical experience, whether operative or otherwise, will please to forward the titles of their articles at once. Any facts observed in connection with the surgery of the abdominal viscera, and especially the kidneys, will be acceptable, but notices of all cases involving new principles of treatment, or illustrating modes of procedure already recognized, are desired. Contributions are requested from every member of the association who has had any service in surgery, and the time being limited, he asks that they may be sent without delay to his address, Atlanta, Georgia.

SCRIBNER'S MAGAZINE.—A notable illustrated article in the February number of *Scribner's Magazine* will be "The Likenesses of Cæsar,'" by Mr. J. C. Ropes. For years Mr. Ropes has been collecting photographs of the head of Julius Cæsar, as it appears in the busts and statues of him. The article is a description of this unique collection, and is finely illustrated with reproductions of the most striking portraits among the number.

THE many friends of Dr. Jno. Thad. Johnson will be pleased to know that he has returned to Georgia and will again locate in Atlanta. He is at present on a visit to his father in Morgan county, but expects to be in Atlanta about the first of February. He was in the city a few days ago, but was very unwell and did not call on his friends, as he would have done but for this indisposition.

REMOVED.—Drs. Taliaferro & Noble have removed their infirmary from 170 South Pryor street to St. Joseph's Infirmary, corner Courtland and Baker streets. This gives them much better quarters than they had before, and their patients can get better nursing here than they could possibly get in a private house. Here they are supplied with the best trained nurses that can be had. We congratulate the gentlemen on the move.

THEY PAID FOR THEIR FUN.—A party of young men in this city decided a few nights ago to have a little fun at the expense of several Atlanta doctors. They thereupon telephoned a number of them to call at one of the hotels and see a very sick man. The night was bitter cold; each physician telephoned for responded at once. They all reached the hotel about the same time. The clerk was surprised; said there was no one sick in the hotel. An investigation revealed the little job put up by the smart young men and they were asked to pay to each doctor five dollars or complaint would be lodged against them at police headquarters. They paid the money, amounting to $40.00, and are now poorer and wiser men.

THE RAID ON THE MEDICAL COLLEGES.—The recent raid on the medical colleges of this city by the police satisfied them that all bodies stolen in Georgia are not sent to Atlanta. Captain Crim, the officer in charge of the raiding party, says he found quite a number of fresh bodies in one of the colleges, but did not find the one he was looking for. He found no fresh subjects in two of the colleges.

COMMENCEMENT EXERCISES.—The Annual Commencement of the Atlanta Medical College will be held in DeGive's Opera House on the night of March 4th. The Annual Address to the graduates will be made by Rev. Henry Clay Morrison, D. D., late of Louisville, Ky., now pastor of the First Methodist Church of this city. Dr. J. C. Johnson will deliver the Valedictory on the part of the class. Mr. Hooper Alexander, of this city, will deliver the prizes.

THE MEDICAL SIGNERS TO THE DECLARATION OF INDEPENDENCE.—The statement in the recent address of the President of the College of Physicians of Philadelphia, that Rush "was the only physician whose name is on that energetic arraignment of the Crown" (the Declaration of Independence) should not pass unchallenged. Josiah Bartlett, of New Hampshire, and Lyman Hall, of Georgia, were successful practicing physicians; Oliver Wolcott, of Connecticut, studied for the profession.—*Boston Medical and Surgical Journal, Jan. 13, 1887.*

